# Drivers of parasite communities in three sympatric benthic sharks in the Gulf of Naples (central Mediterranean Sea)

**DOI:** 10.1038/s41598-022-14024-0

**Published:** 2022-06-15

**Authors:** Mario Santoro, Bruno Bellisario, Valentina Tanduo, Fabio Crocetta, Marialetizia Palomba

**Affiliations:** 1 Department of Integrative Marine Ecology, Stazione Zoologica Anton Dohrn, Villa Comunale 1, 80121 Naples, Italy; 2 Department of Agriculture and Forest Sciences, University of Viterbo, Via San Camillo de Lellis Snc, 01100 Viterbo, Italy

**Keywords:** Ecology, Zoology

## Abstract

Sharks play a key role in the functioning of marine ecosystems and maintenance of trophic web balance, including life cycles of parasites co-occurring in their habitats. We investigated the structure of parasite communities of three sympatric shark species (*Etmopterus spinax*, *Galeus melastomus*, and *Scyliorhinus canicula*) and explored both the influence of host features in shaping the communities and their role as biological indicators of environment stability in the Gulf of Naples (central Mediterranean Sea), a geographical area characterized by strong anthropic pressure. Parasites found were all trophic transmitted helminths with a complex life cycle, except *Lernaeopoda galei*, that is a ecto-parasite copepod. Communities were all similarly impoverished with 4–5 component species and low values of species richness and diversity. Higher abundance of cestode larvae of the genus *Grillotia* was found in *G. melastomus*, although their dominance in all host species suggests that the three sharks have a similar role as intermediate/paratenic hosts in local food webs. Similarly, high abundance of *Grillotia* larvae could also suggest the occurrence of high abundance of largest top predators in the area. Host morphological (fork length in *S. canicula* and *G. melastomus* and body condition index in *G. melastomus*) and physiological (sex and gonadosomatic and hepatosomatic indices in *S. canicula*) variables were differently correlated to parasite community structures depending by host species. Potential reasons for the present impoverished parasite communities are discussed.

## Introduction

Parasites are ubiquitous inhabitants of most ecosystems, occurring in all food webs at all trophic levels^1–4^. In the marine realm, the majority of them are trophic transmitted and exhibit a complex life cycle, requiring a number of intermediate/paratenic and definitive invertebrate and vertebrate hosts to reach their adult stage^1–3^. As these hosts include obligate zooplanktonic and benthic taxa, the study of parasite communities often allows to obtain information on potential preys and predator–prey interactions more efficiently (quickly and cheaper) than proper studies of food habits and trophic position of their hosts, especially in deep marine habitats^2–5^. Moreover, parasites may also provide information about health status and/or deterioration of ecosystems. Perturbations in ecosystem structure and function affect food web topology, and thus also impact upon parasite transmission, modifying communities in composition and abundances^2,4,6–11^. Finally, parasites are even able to impair behaviour, physiology, and morphology of their host individuals from species to population, community, and ecosystem level^12,13^. Therefore, the incorporation of parasite communities in the study of marine food webs is essential to understand predator–prey interactions^3,12^.

Sharks are apex predators and act as key elements with high structural importance within the ecosystem, serving as important links between diverse components of the marine trophic webs^14^. Studies showed that the loss of apex predators such as sharks caused inevitably changes in the intermediate levels of food webs^15,16^, with potential repercussion on trophic interactions and transmission dynamics of parasites with complex life cycles. As such, parasite communities of sharks could be used as useful indicators of biodiversity, food web structure, and environmental stress^2,5,13,17^. Among sharks living in the north-eastern Atlantic and Mediterranean Sea, *Scyliorhinus canicula* (Linnaeus, 1758), *Galeus melastomus* Rafinesque 1810, and *Etmopterus spinax* (Linnaeus, 1758) are carnivorous generalist occupying the mid-high marine trophic levels, thus playing not only an important role as predators, but also serving as prey for largest taxa^18–22^. Given the key position in trophic food webs, they also host a wide variety of heteroxenous parasites; despite of that, comparative analyses of their parasites communities only exist from the Balearic Sea (north-western Mediterranean Sea)^18–20^.

Using these studies as an excellent starting point, we performed a comparative work in the Gulf of Naples (central Mediterranean Sea), a geographical area where these shark species are commonly encountered and that is generally characterized by strong anthropic pressure^23–25^. In particular, this study aimed to: (i) investigate the structure of parasite communities in the three species when living in sympatry; (ii) explore the influence of host features (physiological and morphological) in shaping the composition and abundance of parasite communities; and (iii) discuss the use of parasite communities as biological indicators of environment stability.

## Materials and methods

### Collection and shark examination

Individuals of three sympatric benthic shark species were collected from the Gulf of Naples. Sampling included 102 *S. canicula*, 91 *G. melastomus*, and 39 *E. spinax*. All specimens were obtained between July and August 2020 in the trawling area between Ischia and Capri Islands (~ 40.575816 N, 13.966513 E), at ~ 400–600 m depth^22^. Specimens were frozen until dissections were performed. They constituted the bycatch of scientific and commercial trawling operations (red shrimps’ and pink shrimps’ fishery activities) held with a commercial fishing vessel equipped with bottom trawl nets (mouth of 3 × 4 m in height and width, respectively; 40 mm mesh size), towed at ~ 2–2.5 kn on muddy bottoms^22,26^. Samplings were approved by the ethics institutional review board of Italian Ministry of Agricultural, Food and Forestry Policies and performed in accordance with the permit n. 0008453 (issued May 15, 2020) by the Italian Ministry of Agricultural, Food and Forestry Policies, the guide for the care and use of animals by the Italian Ministry of Health, and the ARRIVE guidelines.

After thawing, the sharks were weighed to the nearest 0.1 g and measured (fork length-FL) to nearest 0.1 cm; sex was determined by gonadal examination. A macroscopic gonadal maturity score (GMS) was recorded to investigate the phase of the reproductive cycle (1 = immature, 2 = maturing, 3 = mature, 4 = resting/regressing) as described in Follesa and Carbonara^27^.

Body condition index (BCI, whole weight/fork length^3^) was calculated as described by Le Cren^28^. The gonadosomatic (GSI, gonad weight/host eviscerated weight × 100) and hepatosomatic indices (HSI, liver weight/host eviscerated weight × 100) were calculated as suggested by Mouine et al.^29^.

### Parasitological study

The skin, gills, mouth cavity, digestive tract, liver, heart, gonads, visceral cavity, and mesenteries of each individual were examined under a dissecting microscope for parasites^10,30^. The musculature of each specimen was cut in thin slices (about 0.5 cm in thickness) and examined under a dissecting microscope for trypanorhynch larvae^22^. For each organ/tissue, ecto- and endo-parasites were collected, counted, washed in physiological saline solution, and preserved in 70% ethanol or frozen (− 20 °C) for morphological and genetic analyses, respectively.

For identification, crustaceans were clarified in 20% potassium hydroxide, and cestodes and trematodes were stained with Mayer’s acid carmine and mounted in Canada balsam or clarified in Amman’s lactophenol. Parasites were studied by light microscope and identified according to the available morphological identification keys^31–36^.

Larval forms of *Anisakis* Dujardin, 1845 spp. and trypanorhynchs were identified or confirmed at the species level using a molecular approach. Genomic DNA from ∼2 mg of middle portion of each larva was extracted using Quick-gDNA Miniprep Kit (ZYMO RESEARCH) following the standard manufacturer-recommended protocol.

For the identification of *Anisakis* larvae, the ITS (Internal Transcribed Spacer) region of rDNA including first internal transcribed spacer (ITS-1), the 5.8S gene, the second transcribed spacer (ITS-2), and ∼70 nucleotides of the 28S gene, was amplified using the primers NC5 (forward; 5′-GTAGGTGAACCTGCGGAAGGATCATT-3′) and NC2 (reverse; 5′-TTAGTTTCTTTTCCTCCGCT-3′)^37^. Polymerase chain reactions (PCRs) were carried out in a 15 µL volume containing 0.3 µL of each primer 10 mµ, 2.5 µL of MgCl_2_ 25 mM (Promega), 15 µL of 5× buffer (Promega), 0.3 µL of DMSO 0.3 mM, 0.3 µL of dNTPs 10 mM (Promega), 0.3 µL (5 U/μL) of Go-*Taq* Polymerase (5 U/μl) (Promega), and 2 *µ*L of total DNA. PCR temperature conditions were the following: 94 °C for 5 min (initial denaturation), followed by 30 cycles at 94 °C for 30 s (denaturation), 55 °C for 30 s (annealing), 72 °C for 30 s (extension), followed by post-amplification at 72 °C for 5 min.

For the molecular confirmation of trypanorhynch larvae, the partial large subunit (lsrDNA, 28S) was amplified using the primers ZX-1 (forward; 5′-ACCCGCTGAATTTAAGCATAT-3′) and 1500R (reverse; 5′-GCTATCCTGAGGGAAACTTCG-3′)^38,39^. PCRs were carried out in a 25 μl volume containing 0.6 μl of each primer 10 µM, 2 μl of MgCl2 25 mM (Promega), 5 μl of 5× buffer (Promega), 0.6 μl of dNTPs 10 mM (Promega), 0.2 μl of Go-Taq Polymerase (5 U/μl) (Promega), and 2 μl of total DNA. PCR temperature conditions were the following: 94 °C for 3 min (initial denaturation), followed by 35 cycles at 94 °C for 30 s (denaturation), 53 °C for 30 s (annealing), 72 °C for 2 min (extension), followed by post-amplification at 72 °C for 7 min.

The successful PCR products were purified using Agencourt AMPure XP (Beckman Coulter), following the standard manufacturer-recommended protocol. Clean PCR products were Sanger sequenced from both strands through an Automated Capillary Electrophoresis Sequencer 3730 DNA Analyzer (Applied Biosystems) using the BigDye® Terminator v3.1 Cycle Sequencing Kit (Life Technologies). The obtained contiguous sequences were assembled and edited using MEGAX v11^40^. Sequence identity was checked using BLASTn^41^.

### Descriptors of parasite community

A component community comprises all parasite species recovered from a sample of a particular host species, while infracommunity refers to the parasites assemblage in one host individual. Prevalence was defined as the number of hosts infected with one or more individuals of a parasite species; parasite species with prevalence higher than 10% in any of the host species will be subsequently referred to as “common”. Abundance was measured as the number of individuals of a particular parasite species in/on a single host regardless of whether or not the host was infected. Mean abundance was measured as the number of individuals of a particular parasite in a sample of a particular host species divided by the total number of hosts of that species examined (including both infected and uninfected hosts)^42^.

The total mean abundance, species richness, Berger-Parker dominance index, and the Brillouin index of diversity were used as overall descriptors of infracommunities for each host species examined^18–20,42^. Total mean abundance was measured as the mean number of individuals of all parasite species, while species richness as the number of parasite species harboured by each shark specimen^42^. Descriptors of community were compared between hosts using the Mann–Whitney U- test.

Parasite species by host specificity were classified as ‘‘specialists’’, defined narrowly as having the bulk of reproducing adults found only in a single host species or having been reported from a single host species, and ‘‘generalists’’, when reported from a variety of related host species^10,43^.

### Multivariate distance matrix regression

Multivariate distance matrix regression (MDMR^44,45^) was used to identify those predictors (**X**) able to explain the multivariate outcome (**Y**) given by the observed distribution of parasites within each shark species. MDMR is a two-step procedure that first computes the pairwise (dis)similarity between samples’ scores along all variables comprising **Y**. Then, pairwise distances are arranged into a symmetric distance matrix (**D**) and the association between **X** and **D** is measured by decomposing the sums of squares of **D** into a portion attributable to regression onto **X** and a portion due to residual. Interestingly, MDMR allows the use of any (dis)similarity metric to quantify the distance between samples (e.g., Euclidean, Bray–Curtis, Manhattan), making MDMR a flexible and robust alternative to methods such as multivariate multiple regression (MMR) and multivariate analysis of variance (MANOVA). Here, morphological (FL and BCI) and physiological (sex, GMS, GSI and HSI) variables^10^ were used as predictors for the parasite’s community. Ecological similarity between individuals of each shark species was measured with the Bray–Curtis index, by using the whole infracommunity data and by removing the contribution of extremely rare species (i.e., those parasites present with a single individual in a single specimen).

We used the square root transformed abundances to retain quantitative information while reducing the influence of dominant species (i.e., *Grillotia*)^46^. MDMR analyses were performed by using the package MDMR^47^ in R^48^, which provides analytical p-values for test statistic and uses a pseudo jack-knife procedure to quantify the conditional effects (δ) of each predictor in **X** (i.e., morphological and physiological traits of sharks) on each outcome variable in **Y** (i.e., the abundance of each parasite species). δ is a relative measure of effect size, with larger values indicating larger effects and vice versa (negative estimates are interpreted as having virtually no effect).

## Results

### Host and parasite data

Biological data (including sex, weight, BCI, GMS, GSI, and HIS) of the three shark species examined for parasites from the Gulf of Naples are reported in Table 1. Basic parameters of infection for each parasite taxon are presented in Table 2.

**Table 1:** Average values (± standard deviation and range in brackets) of morphological and physiological variables of *E. spinax*, *G. melastomus*, and *S. canicula* examined for parasites from the Gulf of Naples.

	*E. spinax* *n* = 39	*G. melastomus* *n* = 91	*S. canicula* *n* = 102
Sex	23 m/16 f	48 m/43 f	51 m/51 f
FL (cm)	26.652 ± 4.913 (14–36.602)	41.267 ± 7.072 (23.503–56)	39.603 ± 6.741 (14–49.511)
Weight (g)	82.331 ± 40.782 (11–180)	195.553 ± 88.462 (39–369)	229.141 ± 179.943 (5–1746)
BCI	0.004 ± 0.0005 (0.005–0.002)	0.003 ± 0.0004 (< 0.001–0.004)	0.003 ± 0.002 (< 0.001–0.028)
GMS	1.411 ± 0.852 (1–4)	2.311 ± 0.902 (1–3)	2.421 ± 0.842 (1–3)
GSI	0.642 ± 0.721 (0.022–2.811)	2.652 ± 3.061 (0.061–13.751)	4.171 ± 4.232 (0.031–16.361)
HIS	15.952 ± 6.244 (4.761–26.871)	3.614 ± 1.965 (1.032–9.452)	8.112 ± 7.433 (2.021–75.852)

Abbreviations: m, male; f, female; FL, fork length; BCI, body condition index; GMS, gonadal maturity score; GSI, gonadosomatic index; HIS, hepatosomatic index.

**Table 2:** Prevalence (P, expressed in percentage) and mean abundance (A ± standard deviation with ranges in brackets) of parasites found in *E. spinax*, *G. melastomus*, and *S. canicula* from the Gulf of Naples.

Parasites	*E. spinax* *n* = 39		*G. melastomus* *n* = 91		*S. canicula* *n* = 102	
	P	A	P	A	P	A
Copepoda						
*Lernaeopoda galei* (A)	–	–	–	–	32.3	0.558 ± 1.022 (0–6)
Trematoda						
*Diphterostomum betencourti* (A)	–	–	–	–	13.7	0.481 ± 1.539 (0–9)
*Otodistomum veliporum* (A)	–	–	1	0.011 ± 0.104 (0–1)	–	–
Cestoda						
*Ditrachybothridium macrocephalum* (A)	–	–	2.1	0.824 ± 7.756 (0–74)	–	–
*Grillotia* sp. (L)	79.4	5.334 ± 6.032 (0–27)	100	181.659 ± 189.129 (1–1421)	94.1	31.509 ± 29.091 (0–188)
*Heterosphyriocephalus tergestinus* (L)	2.5	0.025 ± 0.161 (0–1)	–	–	–	–
*Sphyriocephalus viridis* (L)	5.1	0.051 ± 0.223 (0–1)	19.7	0.362 ± 0.875 (0–4)	–	–
Nematoda						
*Anisakis pegreffii* (L)	–	–	–	–	0.9	0.009 ± 0.099 (0–1)
*Anisakis physeteris* (L)	12.8	0.128 ± 0.338 (0–1)	4.3	0.044 ± 0.206 (0–1)	4.9	0.049 ± 0.217 (0–1)

Abbreviations: (A), adult parasites; (L), larval parasites.

A total of 15 larvae of *Anisakis* spp. (Rhabditida: Anisakidae) were found and all molecularly identified at species level according to the obtained sequences (850 base pairs—bp) at the ITS region of the rDNA. One and 14 larvae were respectively assigned to the species *Anisakis pegreffii* Campana-Rouget & Biocca, 1955 and *Anisakis physeteris* (Baylis, 1923), showing 100% identity with the sequences of *A. pegreffii* and *A. physeteris* previously deposited in GenBank (accession numbers: MF422221–MF422222 and MF668924–MF668926).

The morphological identification of trypanorhynchs was confirmed on a subsample of 70 larvae, according to the obtained sequences (~ 1600 bp) at the 28S gene of the rDNA. A total of 60, 5, and 5 larvae were respectively assigned to *Grillotia* Guiart, 1927 sp., *Heterosphyriocephalus tergestinus* (Pintner, 1913) Dallarés, Carrassón & Schaeffner, 2016 and *Sphyriocephalus viridis* (Wagener, 1854) Pintner, 1913 showing ~ 100% identity with the sequences of *Grillotia* sp., *H. tergestinus* and *S. viridis* previously deposited in GenBank (accession numbers: MW838236, KX570647, and FJ572940, respectively).

Representative sequences here obtained were deposited in GenBank with the accession numbers OM279537 (*A. pegreffii*), OM279534–36 (*A. physeteris*), ON427560–61 (*Grillotia* sp.), ON427564-65 (*H. tergestinus*), and ON427562–63 (*S. viridis*).

Overall prevalence of infection for parasites was 82%, 95.1%, and 100% respectively in *E. spinax*, *S. canicula*, and *G. melastomus*. A total of 20,186 individual parasites belonging to nine taxa (four in *E. spinax* and five each in *G. melastomus* and in *S. canicula*) were identified in the three host species. Only larval forms of a cestode of the genus *Grillotia* (Trypanorhyncha: Lacistorhynchidae) and of the nematode *A. physeteris* were found in all host species; larval forms of the cestode *S. viridis* (Trypanorhyncha: Sphyriocephalidae) were found in two hosts (namely *E. spinax* and *G. melastomus*). In all sharks, the most prevalent and abundant taxon was a *Grillotia* species. An ectoparasite copepod, *Lernaeopoda galei* Krøyer, 1837 (Siphonostomatoida: Lernaeopodidae) was only found from the peri-genital skin of *S. canicula*. All endo-parasites were obtained from the lumen of the gastrointestinal tract, except the larvae of *A. physeteris*, encysted into the wall of stomach and intestine.

The predominant group of parasites with respect to species diversity was the Cestoda (four species), followed by Trematoda and Nematoda (two species each), and Copepoda (one species). Regarding the parasite taxa, the local parasite fauna showed almost similar representation of adult parasite stages [four species: one copepod *L. galei*, two tremadodes *Diphterostomum betencourti* (Monticelli, 1893) Odhner, 1911 (Plagiorchiidae: Zoogonidae) and *Otodistomum veliporum* (Creplin, 1837) Stafford, 1904 (Plagiorchiidae: Azygiidae), and one cestode *Ditrachybothridium macrocephalum* Rees, 1959 (Diphyllidea: Echinobothriidae)] and larval parasite stages [five species: two nematodes *A. pegreffii* and *A. physeteris* and three cestodes *H. tergestinus* (Trypanorhyncha: Sphyriocephalidae), *Grillotia* sp., and *S. viridis*]; however, larval forms dominated numerically the parasite communities of all shark species. In particular, all 216 parasite specimens found in *E. spinax* were larval forms of endo-parasites. Out of the 16,644 parasite specimens found in *G. melastomus*, 76 (0.5%) were adult forms and 16,568 (99.5%) were larval forms. Out of the 3326 parasite specimens found in *S. canicula*, 106 (3.2%) were adult forms and 3220 (96.8%) were larval forms. Out of nine parasite taxa identified, only two (*L. galei* and *D. betencourti*) were considered to be specialist species.

### Descriptors of parasite community

In all shark species, the species richness ranged from one to three, with the maximum number of parasite species observed in a single individual of *E. spinax*, in three individuals of *G. melastomus*, and in four individuals of *S. canicula*. Most individuals of all shark species were infected with a single species of parasite: 66.6% of *E. spinax*, 75.5% of *S. canicula*, and 77% of *G. melastomus*, respectively.

Descriptors of parasite infracommunities for all shark species are listed in Table 3. Only five parasites were considered common, with larval stages of *Grillotia* found in all hosts, *S. viridis* found only in *G. melastomus*, *A. physeteris* found only in *E. spinax*, and *L. galei* and *D. betencourti* found only in *S. canicula*. *Scyliorhinus canicula* and *E. spinax* showed the highest average level of infracommunity diversity indices (Table 3), although their differences were not significant (Mann–Whitney U-test, U < 1858.3 and *p* > 0.06 in all cases). Conversely, *G. melastomus* showed lower diversity values on average, being significantly different from *S. canicula* (Mann–Whitney U-test, U > 2,837.5 and *p* < 0.001 in all cases) but not when compared with *E. spinax* (Mann–Whitney U-test, U < 1418.5 and *p* > 0.7 in all cases).

**Table 3:** Average values (± standard deviation) and range (values in brackets) of measured parameters of parasite infracommunities found in *E. spinax*, *G. melastomus*, and *S. canicula* from the Gulf of Naples.

	*E. spinax* *n* = 39	*G. melastomus* *n* = 91	*S. canicula* *n* = 102
Total mean abundance	5.538 ± 3.768 (0–27)	182.901 ± 111.251 (1–1421)	32.607 ± 18.029 (0–188)
Species richness	1.25 ± 0.508 (1–3)	1.274 ± 0.517 (1–3)	1.546 ± 0.629 (1–3)
Berger-Parker	0.951 ± 0.102 (0.667–1)	0.994 ± 0.036 (0.656–1)	0.958 ± 0.071 (0.6–1)
Brillouin index	0.076 ± 0.154 (0.231–0.567)	0.017 ± 0.072 (0.014–0.667)	0.103 ± 0.141 (0.037–0.481)

### Predictors of infracommunity similarity

Morphological and physiological traits were able to significantly predict the compositional similarity of parasite infracommunity in all three shark species, with the only exception of *E*. *spinax* (Table 4), also showing a negligible contribution of extremely rare species in MDMR model outcomes (Table 4). When considering each predictor, our results showed that both morphological traits (FL and BCI) were significantly (albeit low) correlated with the infracommunity structure of *G*. *melastomus*, while BCI did not correlate in *S*. *canicula* (Table 4). Overall, we did not find a significant effect of sex in *G*. *melastomus* and *E*. *spinax*, while males in *S*. *canicula* showed a similar pattern of parasitic community structure (Table 4). When considering the remaining physiological traits, the MDMR model showed a significant effect of GSI and HSI only for *S*. *canicula*, while GMS did not show any significant effect (Table 4).

**Table 4:** Results of MDMR analyses relating the pairwise compositional similarity of each shark species based on the abundance (square root transformed) of parasites with morphological (FL, weight, and BCI) and physiological (Sex, GSI, HSI, and GMS) variables.

		MDMR Statistic	*df*	Pseudo R^2^	*p*
Omnibus	*Etmopterus spinax*	0.673 (0.621)	7	0.402 (0.383)	0.066 (0.088)
	*Galeus melastomus*	0.815 (0.814)	6	0.449 (0.448)	< 0.002 (< 0.002)
	*Scyliorhinus canicula*	0.571 (0.671)	6	0.364 (0.374)	< 0.002 (< 0.002)
FL	*Etmopterus spinax*	0.047 (0.044)	1	0.029 (0.027)	0.432 (0.458)
	*Galeus melastomus*	0.096 (0.096)	1	0.053 (0.053)	0.002 (0.002)
	*Scyliorhinus canicula*	0.069 (0.072)	1	0.044 (0.044)	< 0.002 (< 0.002)
BCI	*Etmopterus spinax*	0.072 (0.069)	1	0.043 (0.043)	0.272 (0.248)
	*Galeus melastomus*	0.053 (0.053)	1	0.029 (0.029)	0.03 (0.024)
	*Scyliorhinus canicula*	0.004 (0.003)	1	0.002 (0.002)	0.682 (0.656)
Sex (m)	*Etmopterus spinax*	0.087 (0.079)	2	0.052 (0.049)	0.51 (0.582)
	*Galeus melastomus*	0.004 (0.004)	1	0.002 (0.002)	0.698 (0.716)
	*Scyliorhinus canicula*	0.086 (0.088)	1	0.055 (0.054)	0.002 (< 0.002)
GSI	*Etmopterus spinax*	0.044 (0.043)	1	0.02627 (0.027)	0.438 (0.476)
	*Galeus melastomus*	0.003 (0.003)	1	0.002 (0.002)	0.706 (0.716)
	*Scyliorhinus canicula*	0.052 (0.052)	1	0.033 (0.033)	0.012 (0.006)
HSI	*Etmopterus spinax*	0.004 (0.057)	1	0.003 (0.003)	0.934 (0.890)
	*Galeus melastomus*	0.032 (0.032)	1	0.018 (0.018)	0.072 (0.082)
	*Scyliorhinus canicula*	0.204 (0.203)	1	0.129 (0.129)	< 0.002 (< 0.002)
GMS	*Etmopterus spinax*	0.163 (0.151)	1	0.097 (0.092)	0.064 (0.054)
	*Galeus melastomus*	0.001 (0.005)	1	0.001 (0.001)	0.964 (0.958)
	*Scyliorhinus canicula*	0.011 (0.011)	1	0.007 (0.007)	0.422 (0.372)

Omnibus refers to the cumulative effect of all predictors on the distance matrix. Values in parentheses refer to the results obtained by including extremely rare parasite species (i.e., those parasites present with a single individual in a single specimen).

### Relationship between predictors and parasite abundance

**Figure 1:**
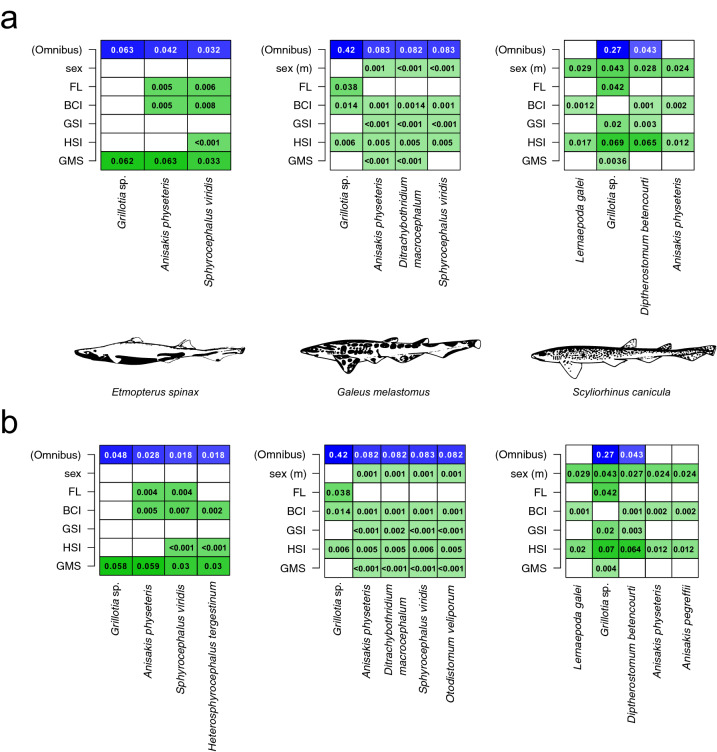
Heatmap of the effect size showing the pairwise effect of morphological (FL and BCI) and physiological (age, sex, GMS, GSI, and HSI) predictors (conditional on the rest of the predictors) on each individual parasites considering the whole infracommunity (**a**) and by removing extremely rare parasite species (**b**, i.e., those parasites present with a single individual in a single specimen). Omnibus (blue cells) refers to the effect size of the entire design matrix on each outcome, while numbers in green cells the pairwise effect size (i.e., the effect of each predictor on each outcome variable, conditional on the rest of the predictors). Sharks in the figure have been redrawn from Compagno^80,81^ and downloaded from https://fishbase.org under a Creative Commons License—CC BY-NC 3.

Overall, a large portion of the MDMR model outcome in each shark species was due mainly to the predictors’ effects on the abundance of *Grillotia* sp., which was much more pronounced on *G*. *melastomus* and *S*. *canicula* (Fig. 1). In this latter species, neither morphological nor physiological predictors had effects on the abundances of *L. galei* and *A. physeteris*, while they had a very limited effect on *D. betencourti* (Fig. 1). Morphological predictors had a very low association with *Grillotia* sp. in *S*. *canicula*, whose abundances seemed to be associated more with physiological traits such as HSI and sex (Fig. 1). Interestingly, in *G*. *melastomus* predictors had a significant influence on the abundances of all parasites, although such effect was much more pronounced for *Grillotia* sp. with FL being the most correlated predictors (Fig. 1). In *E*. *spinax*, the physiological predictor GMS mostly contributed to the abundances of all parasites (Fig. 1).

## Discussion

With the sole exception of *L. galei*, an ecto-parasite copepod found at least in 14 shark and two skate taxa (see https://shark-references.com/species/parasite-hosts-list/L), all the species found were trophic transmitted helminths with a complex life cycle. The present results point out on the evident trophic links between the diet of the host and the parasite occurrence and infection trends^5,49^. In agreement with that, studies on the feeding ecology of *E. spinax*, *G. melastomus*, and *S. canicula* showed that they are carnivorous generalist preying on a great variety of deep-sea invertebrates and fishes, with differences mainly related to host biological features (i.e., ontogenetic changes and gender) and abiotic factors (i.e., sampling season and geographical area). However, while the diet of *E. spinax* and *G. melastomus* mainly consists of decapod crustaceans, cephalopods, and fishes^50–52^, the diet of *S. canicula* consists of more diverse benthic preys, with polychaetes being prevalent in some geographic areas^53–55^.

Among the common helminth species found, a three and four or more host life-cycle has been respectively proposed for trypanorhynch cestodes of the genera *Grillotia* and *Sphyriocephalus* Pintner, 1913, with copepods and molluscs acting as first and second intermediate hosts, predatory fishes as third intermediate or paratenic hosts and elasmobranch species as definitive hosts^35^. The finding of larval stages only of both species suggests that the investigated sharks could acquire the infection by ingestion of both cephalopods and fishes, serving as intermediate/paratenic hosts for these parasite species. Moreover, the different values of abundance of *Grillotia* specimens among the three host species could suggest different amount of intermediate hosts in their diet. For instance, among the shark species studied from the north-western Mediterranean, a *Grillotia* infection was linked to the consumption of mycthophid fishes only in *G. melastomus*, as they were the dominant prey in this host species^18^; however, at the present no evidence exists that mycthophid fishes may play a role in the transmission of the infection.

Regarding the anisakid nematodes here detected, sharks are considered accidental or dead-end hosts of *Anisakis* species. Cetacean species of the families Physeteridae Gray, 1821 and Kogiidae Miller, 1923 represent the main definitive hosts for *A. physeteris* (see^56,57^). Larval stages of *A. physeteris* have been found sporadically in fish species, with low infection rates^56,58,59^, while a recent study suggests the importance of the deep-sea cephalopod *Histioteuthis bonnellii* (Férussac, 1834) as intermediate host in the life cycle of this parasite^60^. The finding of all nematode larvae of the species *A. physeteris*, except a single specimen molecularly identified as *A. pegreffii* (a common parasite of Delphinidae Gray, 1821), strongly supports that the benthonic deep habitat may represent an important food webs system for larvae of *A. physeteris* as previously suggested^56,60^.

*Diphterostomum betencourti* was the only common parasite found as adult in this study. According to Bray and Moore^32^, this trematode is known in *S. canicula* and *S. stellaris* from the Atlantic coast of Belgium, England, France, and Spain, and thus it represents a new geographical record for the Mediterranean Sea. Life cycle for species of the genus *Diphterostomum* Stossich, 1904 has been elucidated only for *D. brusinae* (Stossich, 1888) Stossich, 1904 and *D. flavum* Gilardoni, Etchegoin, Cribb, Pina, Rodrigues, Diez & Cremonte, 2020, two species infecting the teleost fishes. Gastropods serve as first intermediate hosts, while gastropods, bivalves, and other sedentary invertebrates—the crinoid *Antedon mediterranea* (Lamarck, 1816) or the polychaete *Kinbergonuphis dorsalis* (Ehlers, 1897)—as second intermediate hosts^61,62^. Saldanha et al.^53^ reported that polychaetes were the main prey found in the gut of *S. canicula* in the deep sea off southern Portugal, although polychaetes were uncommon preys in similar studies from other geographical areas^51,55^. It has been suggested that the variation in the prey importance could be related to the availability of different benthic assemblages among geographical areas or depths^50,51,55^. Unfortunately, no studies on the feeding ecology of *S. canicula* are available from the Gulf of Naples. The occurrence of *D. betencourti* as common species only in *S. canicula* suggests that polychaetes and/or sedentary invertebrates could represent a discrete part of the diet of this shark species in the study area, supporting the view that the availability of different benthic assemblages might influence the structure of trophic helminth community of the host^63,64^.

The present parasite communities (at both component and infracommunity level) of the three sympatric shark species were all similarly impoverished with 4–5 component species, low species richness ranging from one to three, and low diversity values. Higher total mean abundance was found in *G. melastomus*, but this was due to a higher number of *Grillotia* larvae, which in turn also dominated the communities of other  shark species, suggesting that they have a similar role in the trophic food web of the Gulf of Naples. The high abundance of infection with larvae of *Grillotia* in the present three sharks’ species could also suggest high abundance of largest top predators in the area^22^. The finding of two additional trypanorhynch larval stages—*H. tergestinus* (Pintner, 1913) Dallarés, Carrassón & Schaeffner, 2016 and *S. viridis*—in *E. spinax* also underlines the role of this small-sized shark species as intermediate/paratenic host for members of trypanorhynch cestodes.

Previous studies have already suggested the general idea that Mediterranean small-medium sized sharks have impoverished parasite communities with respect to conspecifics from European Atlantic waters, and that the low values of species richness and diversity coupled with high values of dominance indices are common to small sized sharks from different geographical areas^65^. For instance, seven and 11 taxa of parasites were found in *E. spinax* from Norwegian^66^ and north-east Atlantic waters of Spain^65^, as well as 10 parasite taxa were found in *S. canicula* from off the coasts of Great Britain^67^. However, qualitative and quantitative differences were also evident when we compared the present results to those of the same hosts from the Balearic Sea^19,20^. In particular, five taxa of parasites were found in 41 individuals of *S. canicula*, with no taxon matching the present taxa^19^; a total of 13 parasite taxa were found in 120 individuals of *G. melastomus*, with only two—*D. macrocephalum* and *S. viridis*—of those found in the present study^19^; and only two taxa of cestodes—*Aporhynchus norvegicus* (Olsson, 1868) Nybelin, 1918 and an unidentified tetraphyllidean—were found in 11 individuals of *E. spinax*^20^. Differences were also found among values of infracommunities^19,20^. In particular, total mean abundance in *S. canicula* from Balearic Sea was higher (ranging from 29.14 to 80.56), while Brillouin’s (ranging from 0.03 to 0.09) and Berger-Parker’s (ranging from 0.96 to 0.99) indices were lower than those found in the present study; in contrast, total mean abundance in *G. melastomus* was lower (ranging from 0.08 to 3) and Brillouin’s index higher (ranging from 0 to 0.11) than our results, with similar value of Berger-Parker’s index^19^. Finally, very similar results were found between the present individuals of *E. spinax* and those from the Balearic Sea except for the total mean abundance that in the present study was lower^20^.

Qualitative and quantitative differences in parasite community of the same fish species coming from different geographical areas and depths are expected because specific abiotic factors may be important drivers influencing the presence/absence and abundance of a parasite species in a specific habitat. For example, the nematode *Proleptus obtusus* Dujardin, 1845 was the most prevalent species (until 100%) in individuals of *S. canicula* from Balearic Sea (sampling from 53 to 68 m depth^19,20^), coast of England (sampling depth not stated^67^), and Portugal (sampling depth not stated^68^). However, it was found only in 19.2% of *S. canicula* studied from the north-eastern Aegean Sea (sampling from 40 to100 m depth^69^), and no individuals of this species were found in the present study. *Proleptus obtusus* Dujardin, 1845 is a parasite involved in the shallow marine food web, and the deep environment (400–600 m depth) from which the present shark species were collected could explain the absence of this nematode^19,70,71^.

Regarding the present impoverished species richness of the parasite communities, a recent study, based on a comprehensive analysis of literature records and including several data from 91 different shark species, suggested that the diet breadth of a shark species measured by the diversity of prey families ingested can be the better predictor of cestode species richness^5^. Since the cestodes represent the most important group of parasites in sharks, this could suggest that the populations of the three shark species here studied may have a restricted diet than the same species from other geographical areas, showing in turn poorer parasite communities. Indeed, it is generally agreed that a host with broader diet is exposed to a greater number of potential intermediate host species, resulting in a greater richness of trophically transmitted parasites when compared to a consumer with narrower diet^72–74^.

Moreover, the parasite communities may be good indicators of environmental disturbance because they reflect the interactions between a possible stressor and either free-living larval stages or populations of their intermediate and final hosts^2,6,8,9,63,64^. In the present case, the Gulf of Naples is strongly affected by overfishing and different kinds of anthropic stressors^23–25^, and thus the poor values of parasite communities here found could suggest an unstable ecosystem with a decrease in biomass and richness of intermediate hosts with subsequently restricted diet in the three present sharks^2,6–9^. Indeed, a recent study from two Mediterranean coastal areas, contiguous between them but with different degree of anthropic pressure and deterioration (i.e., Gulf of Naples *versus* Gulf of Salerno), reported that the parasite communities of salema *Sarpa salpa* (Linnaeus, 1758) and white seabream *Diplodus sargus* (Linnaeus, 1758) responded differently to specific biological factors depending by locality, thus highlighting how environmental conditions may exert a strong influence on parasite infracommunities stressing different degrees of deterioration between sampling areas, with lower values of parasite communities found in the Gulf of Naples^10^. Similar differences were described by Derbel et al.^7^ comparing parasite communities of teleost fish from unprotected and protected areas of south-eastern coast of Tunisia.

Morphological and physiological variables were differently correlated to parasite community structure depending on shark species. Some morphological traits (i.e., FL in *S. canicula* and *G. melastomus*, and BCI in *G. melastomus*) were significantly correlated (albeit to a different extent) with parasite abundance. This could be explained by the ontogenetic changes in fish diets, suggesting cumulative infection with age, in particular for larval forms which accumulate in muscles, i.e., *Grillotia* sp.^22,71^. Indeed, it has been documented how individuals of these shark species, increasing in body length, also increase their consumption of cephalopods and mesopelagic fishes^50^, which could represent the main intermediate hosts for trypanorhynch cestodes that dominated numerically the parasite communities in all shark species (i.e., *Grillotia* sp.). Present results were congruent with those of Dallarés et al.^20^ from the north-western Mediterranean, where significant positive relationships with host size were detected at least for the total parasite abundance and the abundance of larvae of *Grillotia adenoplusia* (Pintner, 1903) Palm, 2004 in *G. melastomus*. The lack of correlation between morphological traits and parasite abundance in *E. spinax* could be explained by the fact that most (77%) of individuals of this shark species were juveniles, in contrast to the other two species which were mostly represented by adult individuals.

Regarding the physiological traits, a significant correlation with the similarity of parasite infracommunities was found for GSI and HSI in *S. canicula*. This significant correlation could be explained by the fact that most (73.5%) of individuals of this species were in reproductive stage, as the GSI is a good indicator of reproductive activity of fish while the HSI is strictly related to the gonadal maturation^75^. The present results suggest that large, sexually mature, individuals increasing their food intake in quality and amount could harbour a high number of trophically transmitted parasite species and individuals because they may also ingest more potential infected intermediate hosts^76,77^. The present hypothesis is supported by Capapé et al.^78^, who reported that both male and female GSI values increased with the total length of specimens in *S. canicula*, and HSI reached significantly high values in adults of both sex. A significant effect of sex on compositional similarity of parasite infracommunity was observed only in males of *S. canicula*, suggesting differences in composition of the diet and abundance of infected prey items among genders. The present result was congruent with Mnasri et al.^54^, who showed that preferred preys of *S. canicula* males from the coast of Tunisia were teleost fishes consumed in higher abundance and occurrence, while females preferred crustaceans. The analysis of the relationship between predictors and parasite abundance confirmed the hypothesis regarding the importance of some specific traits, namely HIS and sex in *S. canicula*, FL in *G. melastomus*, and GMS in *E. spinax*.

In conclusion, this represents the second comparative study of parasite community of Mediterranean shark species, being the only data for this basin known from the north-western Mediterranean^18–20^. Depending by host species, both morphological and physiological traits were able to predict the features of parasite communities at component and infracommunity levels. Impoverished parasite communities were found in the three shark species investigated (i.e., *E. spinax*, *G. melastomus* and *S. canicula*). This latter finding could be related to the restricted host diet and/or abiotic factors and decrease in prey biomass and richness resulting in lower probabilities of parasite transmission^2,5–9,63,64,79^ in the Gulf of Naples, an area characterized by overfishing and anthropic pressure^23–25^. Indeed, in agreement to that demonstrated previously^5,63,64^ the parasite fauna of deep-sea fish reflect their diet and the probability of infection and their rates are directly linked to the abundance and richness of free-living prey (i.e., intermediate/paratenic hosts).
